# Osthole inhibits proliferation and induces apoptosis in BV-2 microglia cells in kainic acid-induced epilepsy via modulating PI3K/AKt/mTOR signalling way

**DOI:** 10.1080/13880209.2019.1588905

**Published:** 2019-03-28

**Authors:** Meng Du, Zheng Sun, Yao Lu, Yu-Zhu Li, Hong-Rui Xu, Chang-Qian Zeng

**Affiliations:** a Department ofMedical College, Dalian University, Liaoning, China;; b Beijing International Travel Health Care Center of Beijing Entry-Exit Inspection and Quarantine Bureau, Beijing, China;; c Neonatal Screening Center, Maternal and Child Health Care Hospital of Dalian, Liaoning, China

**Keywords:** Herbal medicine, proliferative inhibition, apoptosis promotion

## Abstract

**Context:** Osthole is a natural coumarin compound most frequently extracted from plants of the Apiaceae family such as *Cnidium monnieri* (L.) Cusson, *Angelica pubescens* Maxin.f., and *Peucedanum ostruthium* (L.). Osthole is considered to have potential therapeutic applications for the treatment of diseases including epilepsy. However, the mechanism of osthole induced-apoptosis in BV-2 microglia cells is not yet clear.

**Objective:** To investigate the molecular mechanisms underlying the effect of osthole on PI3K/AKt/mTOR expression in kainic acid (KA)-activated BV-2 microglia cells.

**Materials and methods:** Optimal culture concentration and time of osthole were investigated by MTT assay. The concentration of osthole was tested from 10 to 400 μM and the culture time was tested from 2 to 72 h. Ultrastructure difference among control, KA and osthole group was analyzed under transmission electron microscope. The mRNA expression of PI3K/AKt/mTOR was investigated using reverse transcription (RT)-PCR and the protein expression was investigated using western blotting and immunofluorescence assay. Apoptosis rate of BV-2 cells between each group was measured by flow cytometry.

**Results:** IC_50_ for cell viability of BV-2 cells by osthole was 157.7 µM. Treated with osthole (140 µM) for 24 h significantly increased the inhibition rate. Pretreatment with osthole inhibited the KA-induced PI3K/AKt/mTOR mRNA and protein expression. The results of flow cytometry analysis showed that the apoptotic rate of osthole group was obviously higher than KA group.

**Conclusions:** Date showed that osthole may be useful in the treatment of epilepsy and other neurodegenerative diseases that are characterized by over expression of PI3K/Akt/mTOR.

## Introduction

Epilepsy is one of the common neurological diseases. It is a brain disease with abnormal function of movement, sensation, consciousness and nerves caused by repeated abnormal discharge of a variety of neurons that are sudden, recurrent, and transient. Epilepsy has a serious impact on patients, families and society (Wang et al. 2012). One of the pathological characteristics of epilepsy is the proliferation of microglia, the decrease and even the complete disappearance of the number of neurons, which leads to the reduction of inhibitory neurotransmitters and the loss of balance between the inhibitory neurotransmitters and excitatory neurotransmitters (Cross and Cavazos 2007; Shapiro et al. 2008).

Osthole (7-methoxy-8-isopentenoxycoumarin, C_15_H_16_O_3_) is a natural coumarin compound most frequently extracted from plants of the Apiaceae family such as *Cnidium monnieri* (L.) Cusson, *Angelica pubescens* Maxin. f., and *Peucedanum ostruthium* (L.). Osthole has been used in traditional Chinese medicine for more than 2000 years, and was first recorded in the book of ‘Shennong's Classic of Materia Medica’, one of the oldest materia medica books. Research shows that osthole exerts many effects including improving cognitive disorder (Ji et al. 2010), anti-osteoporotic (Zhang et al. 2007, 2016), antioxidant (Yan et al. 2017), anti-asthmatic (Wang et al. 2017), antidiabetes (Alabi et al. 2014) and anti-seizure (Luszczki et al. 2009).

Phosphatidylinositol 3-kinase (PI3K)/Akt signal transduction plays an important role in cell growth via inhibition of apoptosis in various types of human cancers (Guerrero-Zotano et al. 2016; Wang et al. 2016; Gao et al. 2017). After processing a cell proliferative up-stream signal mediated by the PI3K/Akt pathway, mTOR phosphorylates and plays a key role in the regulation of cell cycle progression, which includes protein synthesis, tumour growth and angiogenesis (Lang et al. 2007; Zhao et al. 2010). Understanding the mechanism of the effect of osthole on PI3K/AKT/mTOR expression of microglia may elucidate important pathways that may be targeted to treat epilepsy. Therefore, the molecular mechanisms of osthole underlying the effect of osthole on PI3K/AKT/mTOR expression in kainic acid (KA)-activated microglia were investigated.

## Materials and methods

### Reagents

Dulbecco's-modifed Eagle's medium (DMEM) was purchased from Gibco (ThermoFisher, Waltham, MA). Foetal bovine serum (FBS) was purchased from Hyclone (GE Healthcare Life Sciences, Logan, UT). KA was purchased from Sigma-Aldrich (Merck KGaA, Darmstadt, Germany) and osthole was purchased from Dalian Mei Lun Biotechnology Co., Ltd. (Dalian, China). Radioimmunoprecipitation assay (RIPA) lysis buffer, 3-(4,5-dimethylthiazol-2-yl)-2,5-diphenyltetrazolium bromide (MTT) and enhanced chemiluminescence (ECL) kit were purchased from Beyotime Biotechnology Co., Ltd. (Shanghai, China). Trizol and one step reverse transcriptase (RT) premix kit was purchased from Solarbio Biotechnology Co., Ltd. (Dalian, China). Rabbit monoclonal anti-mouse PI3K antibody (Cat. no. 4257), rabbit monoclonal anti-mouse phospho-AKT antibody (Cat. no. 4060), Rabbit monoclonal anti-mouse mTOR antibody (Cat. no. 2983) and rabbit anti-mouse β-actin (Cat. no. 4970), goat anti-rabbit IgG-HRP second antibody (Cat. no. 7074) were purchased from Cell Signaling Technology, Inc. (Danvers, CO). Rabbit monoclonal anti-mouse PI3K antibody (Cat. no. Sc-1637) for Immunofluorescence assay was purchased from Santa Cruz Biotechnology (Santa Cruz, CA). Goat anti-rabbit IgG H&L (Cy3^®^) preadsorbed antibody (Cat. no. Ab6939) was purchased from Abcam (Cambridge, UK). Annexin V Apoptosis kit was purchased from Sangon Biological Engineering (Shanghai, China).

### Cell culture

The BV-2 microglial cells were gifts from Professor Jinyan Wang (Chinese Medical University, Liaoning, China) and were grown in DMEM supplemented with 10% FBS, 100 U/mL penicillin and 100 µg/mL streptomycin at 37 °C in a humidified incubator with 5% CO_2_. The cells were passaged every 3 or 4 d while growing to 80% confluence.

### Cell treatment

Osthole was dissolved in dimethyl sulphoxide (DMSO) and mixed with culture medium to different concentrations. KA was diluted in water to 10 mM in advance and then diluted in culture medium to a concentration of 100 µM. Cells cultured in DMEM without any treatment were served as controls. For the KA group, BV-2 cells were stimulated with KA for 2 h. For the osthole group, cells were pretreated with osthole for a suitable time prior to stimulation with KA.

### *MTT assay for optimal culture concentratio*n

According to the manufacturer’s instructions of MTT cell proliferation and cytotoxicity assay, BV-2 cells were plated at a density of 1 × 10^4^ cells/well in a 96-well plate and cultured in the humidified incubator for adhesion. Then the cells were treated with various concentrations of osthole from 10 to 400 μM for 24 h. After incubation, each well was added with 20 µL MTT [5 mg/mL, in phosphate-buffered saline (PBS)] and incubated at 37 °C for 4 h. The medium was removed and 150 μL DMSO was added to dissolve formazan. The optical density (OD) at 490 nm was measured by a SpectraMax Plus384 Microplate Reader (Molecular Devices, Sunnyvale, CA). The cell viability was calculated according to the following formula: Cell viability (%) = (OD_osthole_/OD_control_) × 100%.

### MTT assay for optimal culture time

Briefly, the KA group was treated with 100 μM KA for 2 h, and then replaced with common medium. For the treated group, BV-2 cells were pretreated with osthole at safe concentration of 140 μM concluded from the above MTT assay prior to stimulate with KA, and then cultured with various times of 2, 4, 6, 8, 16, 24, 48 and 72 h. The inhibition rate was calculated according to the following formula: Inhibition rate (%) = [1−(OD_osthole_/OD_KA_)] × 100%.

### Cell ultrastructure analysis

Ultrastructure difference among control, KA and osthole group was analyzed under a transmission electron microscope (Tecnai G2 20, FEI). The cells were harvested and fixed with 2.5% glutaraldehyde in a 0.05 M phosphoric acid buffer (pH 7.4) at 4 °C for 2 h. Fixed samples were washed with 0.1 M phosphoric acid buffer three times and then fixed with 1% osmium acid at 4 °C for 2 h. Washed three times with phosphoric acid, the samples were dehydrated in a graded ethanol series and embedded in Epon 812 followed by cutting into ultrathin sections. Ultrathin sections were stained in aqueous 1% (w/v) uranyl acetate and lead citrate before photographed.

### Flow cytometry

After 24 h of culture, cells were resuspended in 195 μL binding buffer at a density of 2 × 10^5^ cells/mL. Annexin V-FITC (5 μL) was mixed gently and incubated at room temperature in the dark for 15 min. Cells were washed with 200 μL binding buffer and then centrifuged at 1000 rpm for 5 min, discarding the supernatant. Cells were resuspended in 190 μL binding buffer. Immediately before analysis by a BD FACSCalibur flow cytometry, PI (10 μL) was added to each sample. A minimum of 10,000 cells within the gated region were analyzed.

### Reverse transcription (RT) – PCR

Total RNA was isolated by Trizol reagent and reversely transcribed into single-stranded cDNA using the one step reverse transcriptase (RT) premix kit. cDNA was amplified via PCR with primers for PI3K, AKt, mTOR and β-actin (Table 1). The product lengths for PI3K, AKt, mTOR and β-actin were 93, 504, 823 and 74 bp, respectively. The following PCR conditions were used as follows: 50 °C for 20 min; 95 °C for 3 min; followed by 40 cycles at 95 °C for 20 s; 57 °C (β-actin), 59 °C (PI3K, AKt), or 60 °C (mTOR) for 30 s; 72 °C for 1 min and 72 °C for 5 min. β-Actin was used as an internal control to evaluate the relative expression of PI3K, AKt and mTOR. The PCR products were separated on a 1% agarose gel and visualized under ultraviolet light following staining with DNA green (Tiandz, Beijing, China). The results were analyzed by Image J software (NIH Image, Bethesda, MD).

**Table 1.** Sequences of primers used for reverse transcription-polymerase chain reaction analysis.

Gene	Primer	Sequences
PI3K	Sense	5′-CTGCTCCGTAGTGGTAGAC-3′
	Antisense	5′-TTCATCGCCTCTGTTGTG-3′
AKT	Sense	5′-ATAACGGACTTCGGGCTGTG-3′
	Antisense	5′-TAGGAGAACTTGATCAGGCGG-3′
mTOR	Sense	5′-CCGCCTTCACAGATACCCAG-3′
	Antisense	5′-AGGGATGCCAAGACACAGTAG-3′
β-Actin	Sense	5′-GCAGGAGTACGATGAGTCCG-3′
	Antisense	5′-ACGCAGCTCAGTAACAGTCC-3′

### Western blotting

BV-2 cells were lysed for 40 min placed on ice in RIPA lysis buffer containing 1% phenylmethanesulphonyl fluoride (PMSF). Lysates were subsequently centrifuged at 12000 rpm for 20 min at 4 °C. Protein concentration was quantified using bicinchoninic acid (BCA) protein concentration determination kit. Equal amounts of sample proteins (30 µg) were electrophoretically separated by a 10% (PI3K and AKt) and 8% (mTOR) SDS-PAGE gel. The gel was subsequently transferred to 0.22 µm polyvinylidene fluoride (PVDF) membranes and then soaked in 10% skim milk for 1 h at room temperature, followed by incubation with primary antibodies (1:1000) overnight at 4 °C. After washing with TBST three times, the membranes were incubated with appropriate secondary peroxidase-conjugated antibodies (1:1,000) for 1 h at room temperature. The results were visualized with an enhanced chemiluminescence kit (Beyotime Biotechnology Co., Ltd., Shanghai, China). Images were captured and analyzed using a ChemiDoc™ XRS + imaging system (Bio-Rad Universal Hood II, Bio-Rad, Hercules, CA).

### Immunofluorescence

BV-2 cells were treated as described above on a cell slide in 24-well plate. Then cells were fixed with 4% paraformaldehyde at 4 °C for 20 min and washed in PBST three times for 5 min followed by incubation with normal goat serum (Beyotime Institute of Biotechnology, Shanghai, China) for 30 min. Cells were subsequently incubated with PI3K/AKt/mTOR primary antibody (1:1000) overnight at 4 °C, followed by incubation with goat anti-rabbit IgG H&L (Cy3^®^) secondary antibody (1:1000) for 30 min at room temperature. After three times washed with TBST, DAPI was added into the well incubating for 5 min and then washed with TBST for three times. Images were captured with an Olympus DP73 (Tokyo, Japan) fluorescence microscope.

### Statistical analysis

Values are expressed as the mean ± standard deviation. All statistics were analyzed using SPSS 20.0 (SPSS, Inc., Chicago, IL). One-way analysis of variance followed by Fisher's least significant difference *post hoc* test was used to calculate the statistical differences between the control and treated samples. *p* Value < 0.05 was considered to indicate statistical significance.

## Results

### Safe concentration of osthole on BV-2 cells

**Figure 1.**
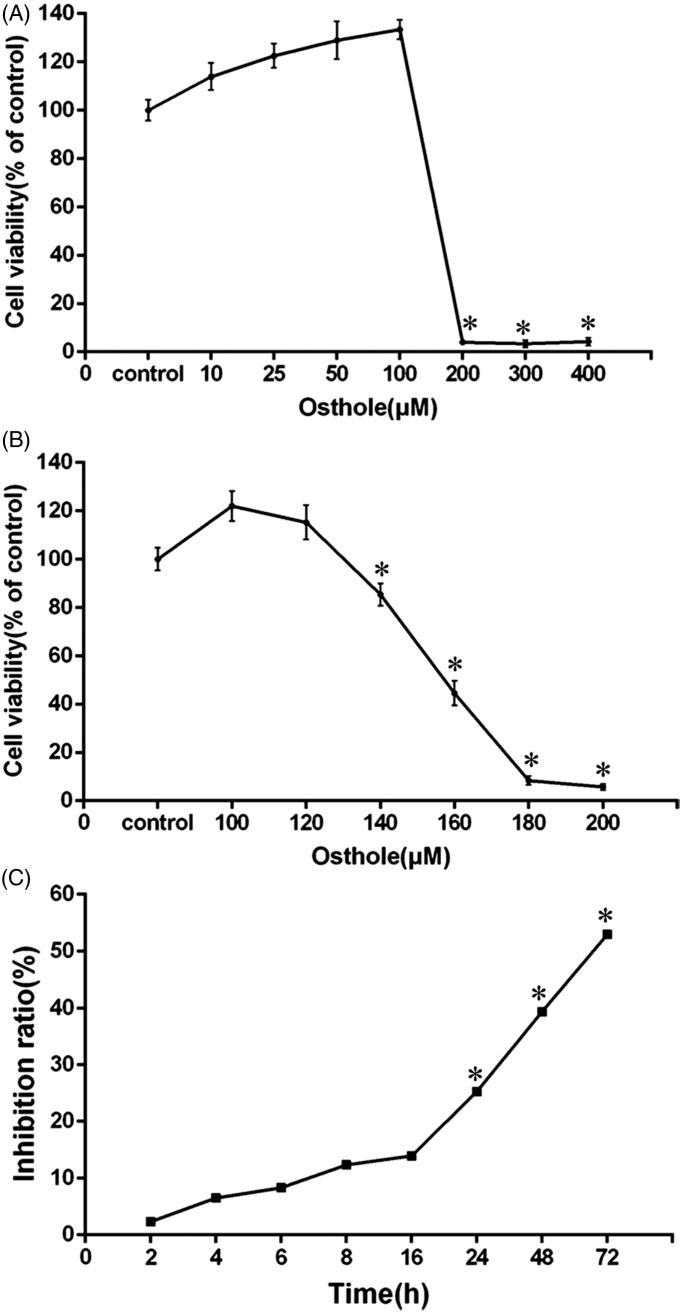
MTT assay for optimal culture concentration and time of osthole inhibiting proliferation and inducing apoptosis in KA-induced BV-2 cells. (A, B) Cell viability between the concentrations from 10 to 400 μM. (C) Inhibition rate of osthole on KA-induced BV-2 cells between the time from 2 to 72 h. Values are expressed as mean ± SD, *n* = 5 per group.

The results of MTT cell proliferation and cytotoxicity assay showed that the cell viability was significantly reduced while the concentration increase from 100 μM (Figure 1(A)). Therefore, the concentrations from 100 to 200 μM with the gradient of 20 were used to culture BV-2 cells. IC_50_ for cell viability of BV-2 cells by osthole was 157.7 µM. As shown in Figure 1(B), cell viability decreased significantly when the concentration increased to 140 μM (*p* < 0.05). Hence, 140 μM was used in the following experiments as the optimum concentration of osthole that inhibiting the proliferation of microglia without destroying cell activity.

### Time-effect on proliferation inhibition of osthole on KA-induced BV-2 cells

As shown in Figure 1(C), there was significant difference in inhibition rate when treated with 140 μM osthole for at least 24 h compared with the KA group (*p* < 0.05). Therefore, 24 h was chosen as the culture time for the following experiments.

### Effect of osthole on the ultrastructure of KA-activated BV-2 cells

**Figure 2.**
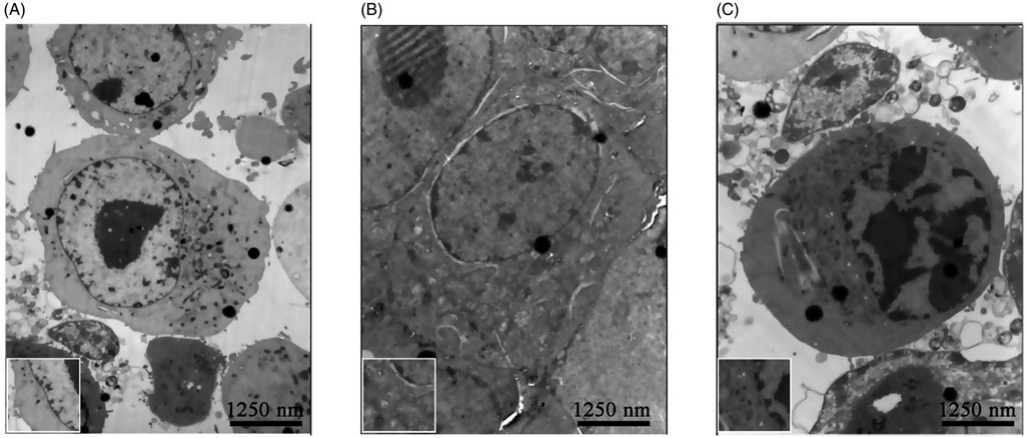
Effect of osthole on the ultrastructure of KA activated BV-2 cells. (A) Ultrastructure of BV-2 cells without any treatment; (B) ultrastructure of BV-2 cells with the treatment of KA for 2 h; (C) ultrastructure of BV-2 cells with the treatment of 140 μM osthole for 24 h before activated with KA for 2 h. Magnification: ×8000 times. Boxed areas shows the complete membrane of BV-2 cells in the control group, the swelled mitochondria in the KA-group and the marginated and contracted chromatin of BV-2 cells in the osthole group. Scale bars, 1250 nm.

The transmission electron microscope images showed that the cell membrane of BV-2 cells in the control group was complete and the cell organelle in the cytoplasm was scarce. The mitochondria were well-structured with clear crista and inner boundary membrane, indicating a static state (Figure 2(A)). The cell body of BV-2 cells in the KA-group became larger and round, forming amoeba-like cells. The organelles were rich in the cytoplasm, the mitochondria swelled and the mitochondrial crista disappeared, indicating that the energy metabolism was very active and showed the characteristic of activation state (Figure 2(B)). In the osthole group, the electron density of BV-2 cells was high, the cell body crinkled, the microvilli decreased, the chromatin marginated and contracted, the nuclear membrane were not clear, the cell organelle in the cytoplasm was blurred and crinkled in the cytoplasm, indicating that it was in a proliferation inhibiting state (Figure 2(C)).

### Effect of osthole on apoptosis of KA-activated BV-2 cells

**Figure 3.**
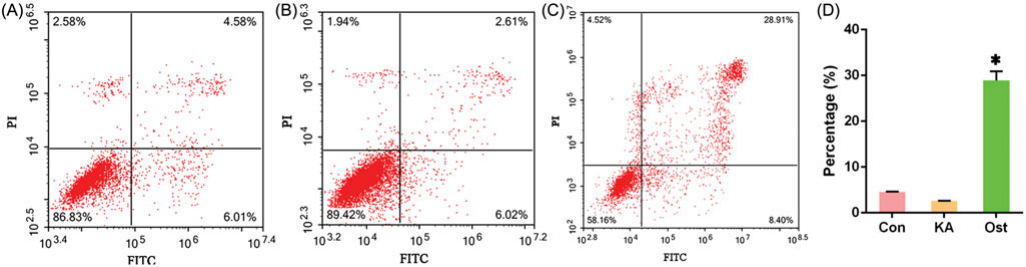
Effect of osthole on apoptosis of KA-activated BV-2 cells. (A) Control group; (B) KA group; (C) osthole group; (D) Histogram of the apoptosis rate evaluated by flow cytometry. *p < 0.05 versus KA group. Con: control; KA: kainic acid; Ost: osthole.

Data of the Annexin V/PI flow cytometry results are shown in Figure 3. Apoptotic rate of control and KA-group was 4.58 ± 0.05% and 2.61 ± 0.03%, respectively, showed that KA can promote the proliferation of microglia BV-2 cells. Apoptotic rate of the osthole group was 28.91 ± 2.01%, showed that osthole can promote the apoptosis of KA-activated BV-2 cells.

### Effect of osthole on PI3K/AKt/mTOR mRNA and protein expression in KA-activated BV-2 cells

**Figure 4.**
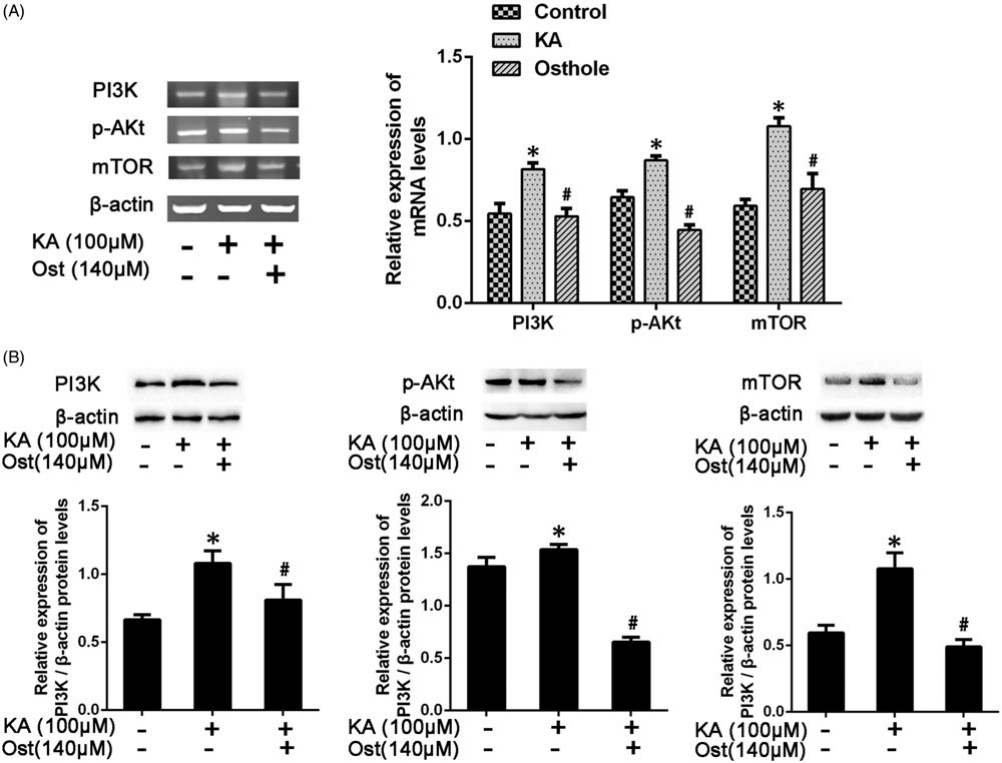
Effect of osthole on the PI3K/AKt/mTOR mRNA and protein expression levels in KA-activated BV-2 microglial cells. The mRNA expression of PI3K/AKt/mTOR was determined by (A) RT-PCR. The protein expression of PI3K/AKt/mTOR was determined by (B) Western blotting. Values are expressed as the mean ± SD, *n* = 3 per group. *p < 0.05 versus control group; #*p* < 0.05 versus KA group. KA: kainic acid; Ost: osthole.

**Figure 5.**
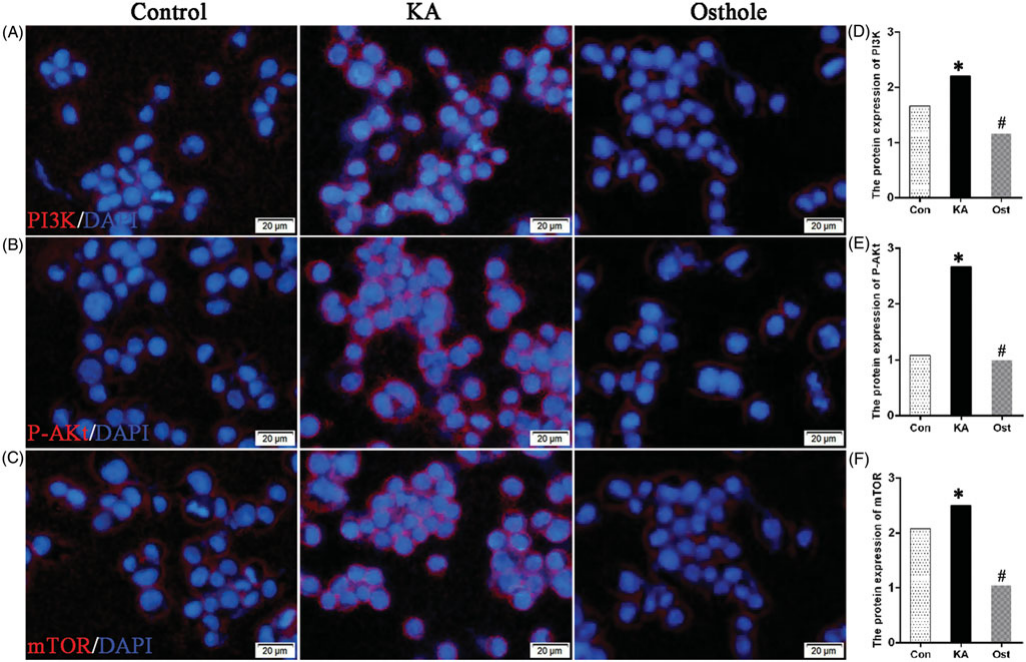
Effect of osthole on the PI3K/AKt/mTOR protein expression levels in KA-activated BV-2 microglial cells. (A–C) Immunofluorescence images of PI3K/AKt/mTOR expression in KA-activated BV-2 microglial cells with nuclear DAPI staining. Magnification: ×200 times. Scale bars, 20 μm. (D–F) Protein expression levels of PI3K, p-AKt, mTOR in KA-activated BV-2 microglial cells quantified by measuring pixel intensity using Image pro-plus software. Values are expressed as the mean ± SD. *p < 0.05 versus control group; #*p* < 0.05 versus KA group. Con: control; KA: kainic acid; Ost: osthole.

The mRNA levels of PI3K/AKt/mTOR were measured by RT-PCR. The protein levels of PI3K/AKt/mTOR were measured by western blotting and immunofluorescence assay. The mRNA expression level of PI3K/AKt/mTOR in the KA-group was significantly higher than that of the control group (*p* < 0.05). Pretreatment with osthole significantly inhibited the KA-induced PI3K/AKt/mTOR mRNA expression (*p* < 0.05) (Figure 4(A)). The results were additionally confirmed by western blotting (Figure 4(B)) and immunofluorescence assay (Figure 5), which exhibited an increased level of PI3K/AKt/mTOR in KA-treated BV-2 cells, and a decreased level in BV-2 cells pretreated with 140 μM osthole for 24 h. The results suggested that osthole inhibits proliferation and induces apoptosis may target signalling pathways involved in PI3K/AKt/mTOR expression in KA-activated BV-2 microglial cells.

## Discussion

As a refractory disease of the nervous system, epilepsy has remained a significant social concern and financial burden globally. Current treatment strategies are almost based on neurocentric mechanisms that have not got a complete victory, raising the need for novel alternative and complementary approaches (Hernan and Holmes 2016; Fulton et al. 2017; Kim et al. 2017). Microglia, the main antigen presenting cell in the central nervous system, accounts for about 7% of the total number of glial cells, which plays an important role in maintaining the stability of the microenvironment of the nerve cells (Colonna and Butovsky 2017). Under normal conditions, microglia is staying in a resting state. When response to abnormal stimulation, microglia is stimulated and activated, rapidly proliferates and migrates to the injured part, mediates the neuroinflammatory reaction, and eventually leads to neuron apoptosis (Dambach et al. 2014; Brifault et al. 2017). Therefore, in the pathological mechanism of epilepsy, there may be mutual promotion and causal relationship among the factors such as neuron apoptosis, neuronal excitability toxicity, microglia proliferation activation and inflammatory mediators. Abraham et al. (2012) found that the microglia in the brain increased two times in KA-induced epilepsy in rat. Microglia inhibitors were found to inhibit the activation of microglia and reduce the susceptibility of seizures. Therefore, the activation and proliferation of microglia is one of the important mechanisms of the progressive development of epilepsy. Inhibition of the activation and proliferation of microglia may be an effective measure to alleviate post epileptic injury.

Osthole is an active constituent extracted from some medicinal plants. It has been reported that osthole exerts its neuroprotective effect on neuronal synapses in Alzheimer's disease (AD) (Li et al. 2016). Li et al. (2016) demonstrated that osthole has neuroprotective effect against cerebral ischemia/reperfusion injury through an anti-apoptotic pathway in rats.

Phosphatidylinositol 3-kinase (PI3K)/Akt signal transduction plays an important role in cell growth via inhibition of apoptosis in various types of human cancers (Riquelme et al. 2016; Zhang et al. 2015; Nandini et al. 2017). The mTOR signalling pathway plays a crucial role in regulating cellular growth and proliferation by directly regulating protein synthesis (Kang et al. 2012). A principal pathway that signals via mTOR is the PI3K/Akt signalling pathway, which is critically involved in the regulation of cell proliferation and survival.

The results of the present study indicated that 140 μM osthole can inhibit the proliferation of microglia without destroying cell activity and 24 h culture time of osthole can significantly inhibit the cell activity of KA-activated BV-2 microglia cells.

Data showed that osthole significantly decreased the mRNA and protein expression of PI3K/Akt/mTOR in KA-activated BV-2 microglia cells. The high apoptosis rate of the osthole group also suggested that osthole can promote the apoptosis of KA-activated BV-2 cells.

## Conclusions

The evaluated parameters indicated osthole inhibits proliferation and induces apoptosis in BV-2 microglia cells in kainic acid-induced epilepsy may be via modulating PI3K/AKt/mTOR signalling way. Osthole may be useful in the treatment of epilepsy and other neurodegenerative diseases that are characterized by over expression of PI3K/Akt/mTOR.
